# Uniform Approximation Is More Appropriate for Wilcoxon Rank-Sum Test in Gene Set Analysis

**DOI:** 10.1371/journal.pone.0031505

**Published:** 2012-02-07

**Authors:** Zhide Fang, Ruofei Du, Xiangqin Cui

**Affiliations:** 1 Biostatistics Program, School of Public Health, Louisiana State University Health Sciences Center, New Orleans, Louisiana, United States of America; 2 Department of Biostatistics, University of Alabama at Birmingham, Birmingham, Alabama, United States of America; University of Texas School of Public Health, United States of America

## Abstract

Gene set analysis is widely used to facilitate biological interpretations in the analyses of differential expression from high throughput profiling data. Wilcoxon Rank-Sum (WRS) test is one of the commonly used methods in gene set enrichment analysis. It compares the ranks of genes in a gene set against those of genes outside the gene set. This method is easy to implement and it eliminates the dichotomization of genes into significant and non-significant in a competitive hypothesis testing. Due to the large number of genes being examined, it is impractical to calculate the exact null distribution for the WRS test. Therefore, the normal distribution is commonly used as an approximation. However, as we demonstrate in this paper, the normal approximation is problematic when a gene set with relative small number of genes is tested against the large number of genes in the complementary set. In this situation, a uniform approximation is substantially more powerful, more accurate, and less intensive in computation. We demonstrate the advantage of the uniform approximations in Gene Ontology (GO) term analysis using simulations and real data sets.

## Introduction

High-throughput gene expression profiling technologies, such as microarray and next generation sequencing, generate expression measurements for thousands of genes simultaneously. One of the most important applications for this technology is in identifying differentially expressed genes across conditions/treatments/tissues. This application often involves comparing the expression of each gene in different conditions/treatments/tissues. To facilitate the interpretation of the results, genes with same annotation attributes, such as those in Gene Oncology (GO) and Kyoto Encyclopedia of Genes and Genomes (KEGG), are often combined into gene sets to infer the involvement of biological processes and pathways in the comparison.

There are many methods and tools for gene set analysis, such as those reviewed and compared previously [1]–[3]. According to [4], these methods can be roughly separated into three categories, “competitive”, “self-contained”, and “hybrid” procedures based on the hypotheses they are testing and the procedures for obtaining significant thresholds. Most of the tools available these days belong to “competitive” category as summarized by Huang *et. al.* [2] and the publication based on this category of methods keep growing more than those based on other categories due to its simplicity [5].

For the “competitive” hypothesis testing category, Fisher's exact test based on a list of significant genes has been widely used because of its simplicity. However, it is based on the dichotomization of genes into significant and nonsignificant groups and the results may vary depending on the threshold for dichotomization. A natural replacement for Fisher's exact test is the Wilcoxon Rank-Sum (WRS) test, which is a rank based non-parametric test for comparing two groups of observations without the assumption of certain distribution [6]. It does not require dichotomization of the genes. WRS has been implemented in many packages and software for both “competitive” and “hybrid” gene set testing. These include the widely used *limma* (R function *geneSetTest*, [7]), *safe* (R function *safe* [8]), and *GOstat* (http://gostat.wehi.edu.au [9]). It has been shown recently that WRS performs better as a gene-set level statistic [3] based on controlled mutual coverage from different gene-set level statistics when the null distribution was obtained using sample permutation.

There are two ways to obtain the null distribution of the WRS statistic, sample permutation and gene permutation. The sample permutation is used in the “hybrid” methods [8], [10]. It is considered to be more desirable than gene permutation in handling correlations among genes. However, sample permutation can run into problems when the number of samples is small. For example, a two group comparison of 4 versus 4 will only have 70 unique permutations, which will not provide a null distribution to generate a p values less than 0.014. This scenario is common for microarray and RNA-seq experiments intended for identifying differential expression. Gene permutation is used in the “competitive” hypothesis testing [4]. Although the exact null distribution can be calculated, it is often too computationally intensive when the numbers of observations in the two comparing groups are large. In practice, a normal distribution is used for approximation as implemented in the standard software packages such as SAS, STATA, Splus, and R [11]. In fact, SPSS and R use exact computation for comparisons with 49 or less total observations at the absence of ties. Bellera *et. al.* showed that normal distribution can approximate the exact distribution well graphically even for relatively small number of observations, such as 9 vs 5. However they did not address the issue of imbalance between the two comparing groups. From their results, it is obvious that the normal approximation for a comparison of 5 vs 5 observations is much better than a comparison of 8 vs 2 observations although the total number of observations is the same in the two scenarios. Buckle *et. al.* [12] showed that a uniform distribution is uniformly better to approximate the null distribution of WRS statistic based on the exact calculations of imbalanced groups with less than 50 observations in each group. In gene set analysis, this imbalance is exacerbated. The small group, the set of genes to be tested, often has less than 10 observations (genes) present in the data set while the complementary group has thousands of observations (genes). In this case the advantage of uniform approximation is expected but the degree is not clear.

In this paper, we compare the uniform approximation and the normal approximation for WRS statistic in the “competitive” hypothesis testing of gene set enrichment for microarrays and RNA-seq data, where the number of genes in the gene set is small while that the number of genes in the complementing group is huge. Our simulation and real data analysis demonstrate that the uniform approximation is a much better approximation for small GO term analysis. It is much more powerful, more accurate, and it requires less computational time.

## Results

### Uniform approximation is much more accurate in extreme cases

Given the fact that the total number of genes is usually huge in GO term tests, it is difficult to obtain the exact p values based on permutation due to limitations of time and computational capacity. Traditional normal approximation is employed in many software packages. Although we cannot obtain exact p values to assess the accuracy of approximation methods due to the computation limitation, we can use some special cases as example to demonstrate the problem of normal approximation. When the local statistics in a gene set are either all greater or all smaller than those in the complement of the gene set and there is no tie, **Table 1** shows p values obtained from the exact permutation, the normal approximation, and the uniform approximation separately. These p values clearly indicate that the normal approximation dramatically underestimates the significance. The uniform approximation is much more accurate.

**Table 1:** Exact and approximate p-values for some extreme cases.

m	3	6	9	12	20
Exact	6.00e-12	7.21e-22	3.64e-31	4.82e-40	2.48e-62
Uniform	7.15e-12	2.46e-21	4.97e-30	3.02e-38	1.21e-58
Normal	1.35e-3	1.11e-05	1.32e-07	1.01e-09	5.06e-15

m, the number of genes in the gene set. Total number of genes is set as 10,000.

### False positive rate is under control for uniform approximation

We used simulation to investigate the false positive rates of two approximation methods. We directly generated local statistics for each gene for WRS test in gene set analysis instead of gene expression levels. The local statistics were randomly drawn from normal distribution *N(3,1)* for gene sets with 5, 10, 15, 20, or 25 genes. The total number of genes inside and outside of the gene set was set as 15,000 to be realistic. We conducted 100 simulations to calculate the rate of false positive calls at three significance levels (0.05, 0.01, and 0.001). The whole process was repeated 50 times to obtain the mean false positive rate (**Table 2**). The results indicate that the mean false positive rates of the uniform approximation are very close to the specified significance level. However, the normal approximation is too conservative for small gene sets at more stringent significance levels (α = 0.01 and α = 0.001), which are often used in practice when multiple testing is applied for testing large number of gene sets.

**Table 2:** False positive rates comparison between uniform and normal approximations to WRS test.

	m	5	10	15	20	25
α = 0.05	Uniform	0.0478	0.0518	0.0494	0.0514	0.0476
	Normal	0.0454	0.0510	0.0492	0.0510	0.0472
α = 0.01	Uniform	0.00915	0.01035	0.00925	0.0099	0.0108
	Normal	0.00595	0.00875	0.00845	0.0093	0.0103
α = 0.001	Uniform	0.00095	0.00125	0.0014	0.0009	0.0011
	Normal	0.0002	0.0007	0.0008	0.0007	0.001

Results are the mean of false positive rate from 50 rounds of simulations. m, number of genes in gene set.

### Uniform approximation is more powerful

**Figure 1:**
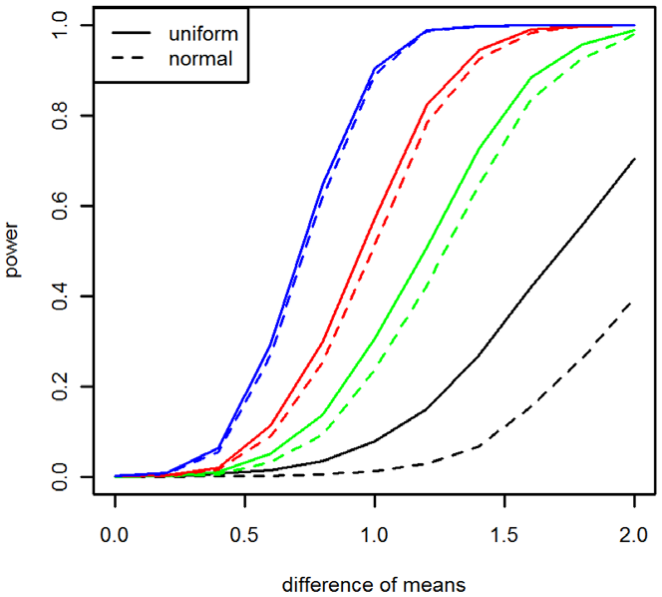
Power comparison between uniform and normal approximations. Black, green, red, and blue lines represent gene sets with 5, 10, 20, and 25 genes, respectively. Significance level is 0.05 with Bonferroni correction. Total number of genes in the experiment were set as 15000.

We also used simulation to compare the power of the two approximation methods. The procedure is the same as that for assessing the false positive except that an effect size δ (from 0 to 2) is added to the mean of the normal distribution from which random numbers were drawn for the genes in the gene set. We conducted 100 simulations to calculate the power at significance level of 0.05 with Bonferroni correction. The whole process was repeated 30 times to obtain the power. The results showed that the uniform approximation has more power than the normal approximation in all settings of gene set size (**Figure 1**). The power improvement for gene sets with 5 genes is dramatic. The improvement for gene sets with 10 genes is still substantial, but the improvement decreases to negligible for gene sets with 25 genes.

### Most GO terms are small in the microarray and RNA-seq datasets

**Figure 2:**
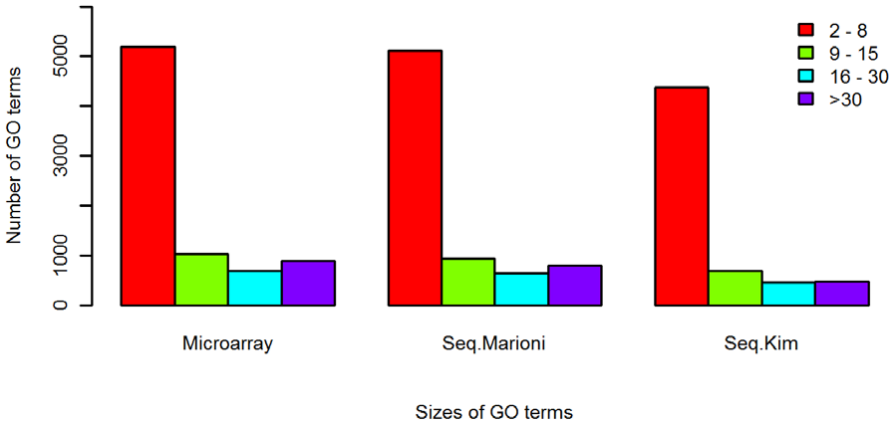
The distributions of the sizes of GO terms in the microarray and RNA-seq datasets analyzed in this paper. Most GO terms are small.

To examine the GO term sizes, we mapped the genes on the microarray of the analyzed array datasets or those detected in the RNA-seq datasets to GO terms. **Figure 2** shows the distributions of the sizes of GO terms. The largest fraction of the GO terms has sizes between 2 and 8. Only a very small fraction has sizes greater than 30. This justifies the need of good approximation to the null distribution of the WRS test statistic.

### Uniform approximation generally gives smaller p values in real data analysis

**Figure 3:**
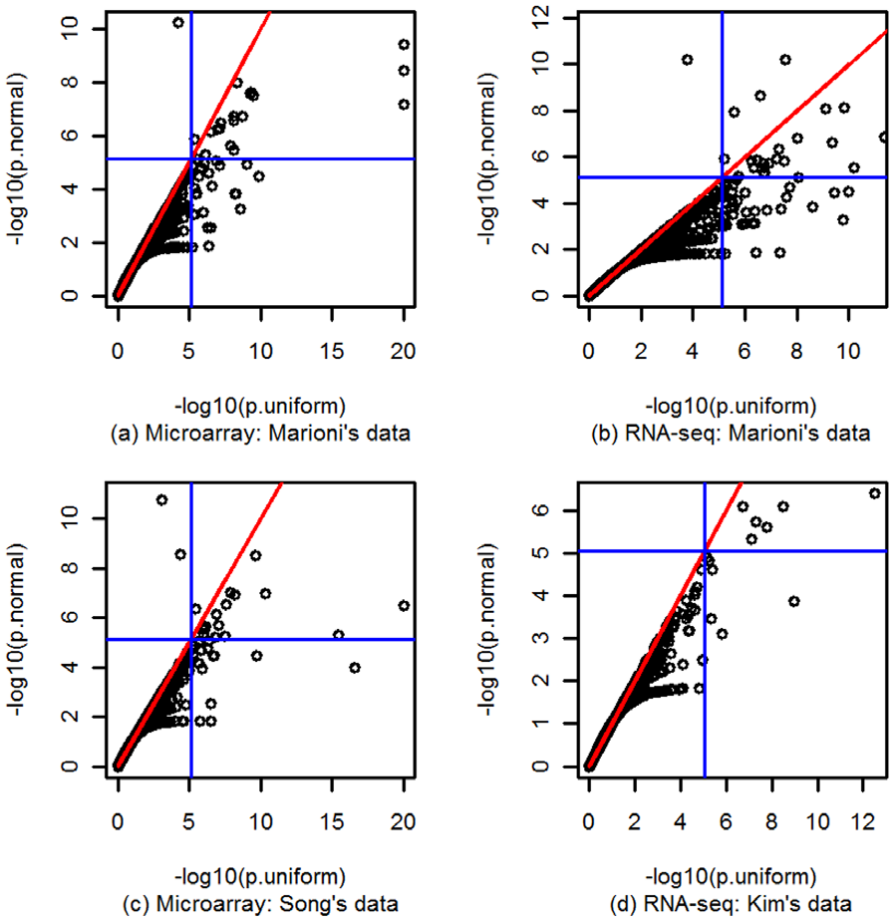
Comparison of p values obtained from normal and uniform approximations. The p values (negative base 10 logarithm) from the normal approximation are plotted against those from the uniform approximation. The red line is the identity line and the two blue lines represent the cut-off p value of 0.05 with Bonferroni correction. Panels (a) and (c) are for the two microarray datasets. Panels (b) and (d) are for the two RNA-Seq datasets.

For the purpose of comparing approximations, we focused on GO terms with 2–30 genes. We examined the p values obtained from analyzing GO terms in the microarray and RNA-seq datasets when the two approximations were used for the null distribution of WRS test. The results showed that the p values are similar between the two approximation methods for GO terms with relatively large p values, such as p values greater than 0.01 (**Figure 3**). However, for GO terms with smaller p values, uniform approximation generally gives smaller p values than the normal approximation for all datasets. For a given p value cutoff, there are substantially more GO terms deemed as significant when uniform approximation is used. For example, at significance level of 0.05 with Bonferroni correction (blue line in the plots), uniform approximation detected 44 significant GO terms in the Marioni microarray data (**Figure 3a**), while the normal approximation only detected 20 of them. Similar results were obtained from the Song microarray data (**Figure 3c**). For the Marioni RNA-seq dataset, the uniform approximation detected 66 significant GO terms while the normal approximation detected only 30 GO terms at the same significance level (**Figure 3b**). Similar results were also obtained from the Kim RNA-seq data (**Figure 3d**) except that the significance level of 0.05 with Bonferroni correction seems too stringent for this dataset and only several GO terms are significant. However, the relationship of the p values from the two approximations remains the same. Note that there are one or two GO terms in (a)–(c), which are significant if normal approximation is used and insignificant if uniform approximation is used. However the sizes of these GO terms are actually large: the one in (a) has size of 30; the one in (b) has size of 28 and the two GO terms in (c) has equal size of 29.

The uniform approximation also reduces the computation time substantially. The computations were carried out using one 2.80 GHz CPU with 8.00 GB installed memory on a Dell Precision T1500 Desktop (64-bit operating system). For the Marioni microarray dataset, the CPU time was 63 seconds for the uniform approximation and 336 seconds for the normal approximation for all GO terms in the microarray data. For the Marioni RNA-seq dataset, the CPU time was 54 seconds and 259 seconds for the uniform and normal approximations, respectively. It is worthwhile to point out that the function “*wilcox.exact*” in *R* package “*exactRankTests*” adopts the Shift-Algorithm [13] to calculate the exact p value, but needs lengthy computation. We used Marioni RNA-Seq data as an example and found that typical CPU times were 16 minutes for wilcox.exact to calculate the exact p value for one GO term with 6 genes and 3.5 hours for another GO term with 26 genes. The computational time for all the 4081 GO terms would be unmanageable. This function returns normally approximated p values if the exact argument is specified as *FALSE*.

### The effect of the local statistic

**Figure 4:**
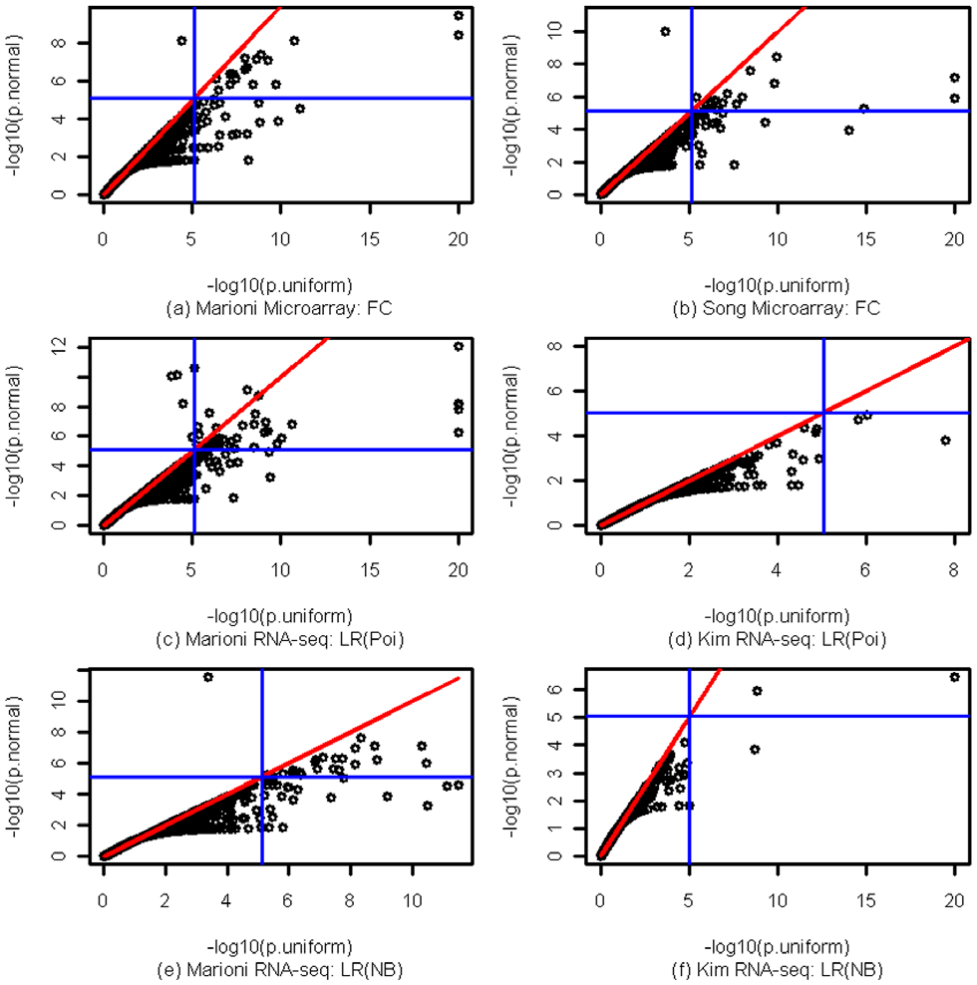
Effect of local statistics on the comparison of the two approximation methods. The plot are of negative base 10 logarithm of the p-value from normal approximation versus that of the p-value from uniform approximation when using fold change (microarray) and log likelihood ratio (RNA-Seq) as local statistics. Panels (a) and (b) are two different microarray datasets. Panels (c) and (d) are the RNA-seq datasets with Poisson assumption. Panels (e) and (f) are the RNA-seq datasets with Negative Binomial assumption.

We also examined the effect of different local statistics on the comparison of the two approximations for the null distribution of WRS test in analyzing the real data. We evaluated fold change, the difference of the mean expression levels (on log2 scale) in liver and kidney samples, for the Marioni microarray data and likelihood ratio (LR) test statistic for the RNA-seq datasets, with the assumption of Poisson (Poi) model or Negative Binomial (NB) model for gene expressions, respectively. The results are shown in **Figure 4**. The results look very similar to those in **Figure 3** except that comparing to **Figure 3(b)**, there are three extra points in **Figure 4(c)** showing apparent smaller p values from the normal approximation and significant but insignificant from the uniform approximation. Two of these points correspond to GO terms with size of 29 while the other point links to a GO term of size 30. These results indicate that the choice of local statistic does not have much impact on the relative performance of the two approximations. The uniform approximation is more powerful.

### Software

We have written a simple R function for the uniform approximation of the null distribution for WRS test. It will be available upon the publication of this paper at http://publichealth.lsuhsc.edu/wilcoxon.html.

## Discussion

Based on both simulation and real data analyses, we demonstrated that, compared with the conventionally used normal distribution, the uniform approximation for Wilcoxon Rank-Sum statistic has better controlled false positive rate, more power, and more accurate p values in gene set enrichment analysis when the number of genes in a GO term is less than 30. When the uniform approximation is used for real datasets analysis, more GO terms are detected as significant. Therefore, the uniform approximation is a better choice for calculating p values for small gene sets should one use WRS test for gene set enrichment analysis in competitive hypothesis testing. For gene sets with 30 genes or more, normal approximation performs well.

In Gene set enrichment analysis, the better approximation to the null distribution of WRS statistic from the uniform distributions lies in the fact that the uniform approximation gives lower tailed probabilities than the normal approximation. Both the sum of independent uniform random variables and the normal random variable have symmetric densities. However, the normal density has longer tails. The difference between two densities becomes negligible when the number of genes in the gene set approaches 30. In fact, by the central limit theorem, the sum of the independent uniform random variables is approximately normally distributed as the numbers of variables is equal or over 30.

Gene set analysis is an important step in understanding the biological processes involved in the differential expression when samples from different treatment/tissues are compared. Different methods and tools for gene set analysis often test different hypotheses as pointed out by Georman and Buhlmann [4]. It has been pointed out that the sample based permutation in the self-contained hypothesis testing and the hybrid procedures are more appropriate in handling correlations among genes and reflecting the natural replication units [1], [4], [14]. However, due to the simplicity and limitation of sample sizes in many microarray experiments, the gene permutation based competitive hypothesis testing procedures are still used more than the sample permutation based procedures in expression microarray data analyses [5]. For RNA-seq data, the sample size is even smaller due to the cost of the technology [15], which limits the use of sample permutation. In this case, the gene permutation for “competitive” hypothesis testing is the only practical way for the gene set analysis. When competitive hypothesis is tested, it is commonly recognized that WRS test is better than the Fisher's exact test or the χ^2^ test. Therefore, studying the appropriate null distribution of WRS statistic is still very much relevant to the current technology applications. A better null distribution will provide more accurate p values that directly affect the significance of pathways and GO terms at a given significance level. The significant pathways and GO terms are often further studied in follow-up research. More broadly, this work is potentially also applicable to gene set analysis in genome-wide association studies and rare variant analysis for identifying pathways and biological processes underlying human diseases.

## Methods

### Approximation to the null distribution of the Wilcoxon Rank-Sum test

Assume a random sample 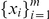 from the population 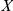 with cumulative distribution function (CDF) 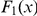, and another random sample 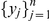 from the population 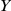 with CDF 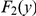, with *m* and *n* as the number of items in X and Y, respectively. Without normality assumption to 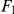 and 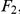 WRS Test is a commonly used in practice to test hypothesis 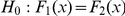 versus 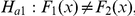 or 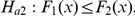 or 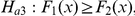 for all 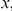 with strict inequality holding for at least one 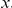 The two-sided alternative hypothesis 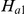 indicates that two population distributions are different, while one-sided hypothesis 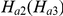 indicates that 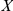 is stochastically larger (smaller) than 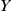 [16]. WRS test uses the sum of ranks of 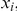 in the combined observations 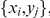 as the statistic 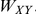 When both *m*, *n* are sufficiently large, the null distribution can be approximated by a normal distribution with mean, 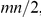 and variance, 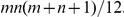 The mean and variance are adjusted when ties occur. The function *wilcox.test* in *R* environment can be used to calculate the exact p value or normally approximated p value (default) of a WRS test. This function will automatically return normally approximated p value should ties occur. WRS test is equivalent to Mann-Whitney test in that 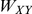 is 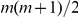 greater than the Mann-Whitney test statistic. Mann-Whitney test statistic is defined as the count of pairs 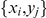 with 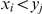 and its value is reported by R function *wilcox.test*.

The normal approximation to WRS test performs well when both *m*, *n* are sufficiently large (greater than 30), but is less accurate when *m* is small and *n* is extremely large. The latter situation is common in gene set analysis, where *m*, the number of genes in a gene set (such as a GO term), is small, such as from 3 to 10, but *n*, the number of genes in the complement of the gene set, is huge (usually *n*>10000 in high-throughput genomic data). In this case, the uniform approximation proposed by Buckle *et. al.* [12] is likely more appropriate. With 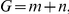 they showed that, as *n* goes to infinity, the null distribution of 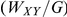 is the same as that of the sum of *m* random samples from uniform distribution in (0, 1). Specifically, if *w* is the observed value of 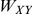 and 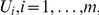 are independent uniform (0, 1) random variables, the probability 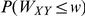 can be approximated by 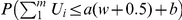, where 0.5 is the continuity correction and
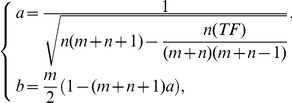
with *TF* being a quantity due to ties (zero if no tie occurs). If there are 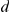 distinct values in the combined observations and that 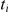 of the 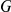 observations are equal to the 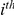 smallest value, 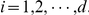 Then 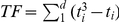 [16].

The distribution of the sum of independent uniform (0, 1) random variables is available in the literature. That is (see, for example [17], for the density and [18] for the cumulative distribution)
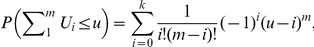
where 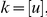 the largest integer not greater than *u* and 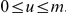 This probability gives an approximation to the one-sided 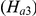 WRS test p value. The p value for the two-sided WRS test (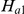) is the twice of the smaller of two one-sided p values and it was used for the real data analyses through out the paper.

### Simulations

For each simulation, we generated two groups of normal data. Group one has *m* random numbers from a normal distribution 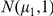 and group two has *n* random numbers from another normal distribution 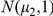. We used fixed total number of genes *G* ( = *m*+*n*) and varied the number of genes in a gene set (*m*) over {5, 10, 15, 20, 25}. The process was repeated 100 times to obtain false positive rate. The simulated false positive rate was then calculated as the percentage of times that the null hypothesis of equal means was rejected by WRS test when 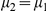. The simulations for assessing false positive rate are as follows.

1. Generate *G* (15000) random numbers from a normal distribution 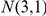.
2. Randomly select *m* simulated data points from 1) as the local statistics in a gene set. Obtain p-values by uniform and normal approximation to WRS test separately for this gene set.
3. Repeat step 2) 100 times and obtain the simulated false positive rate, with significance levels of 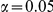, 0.01, and 0.001.

We repeated the process from 1) to 3) 50 times to account for the randomness in the simulation and calculated the average false positive rates. For assessing power, we set the effect size δ = (0, 0.2, 0.4, 0.6, 0.8, 1, 1.2, 1.4, 1.6, 1.8, 2). For each gene set size m = (5, 10, 15, 20, 25).

1. Generate a group of m random number from *N*(3, 1) and another group of (G – m) random number from *N*(3+δ,1)
2. Obtain the p values for the data in 1) by uniform and normal approximation separately.
3. Repeat steps 1) – 2) 100 times and calculate the simulated power of each approximation method as the percentage of times that the p value is lower than 0.0005.

We repeated the procedure 30 times and calculated the average simulated power. Note the choice of normal mean in step 1) for both simulation procedures is arbitrary and should not affect the simulation results given the nonparametric nature of the WRS test.

### Real datasets and processing

**Figure 5:**
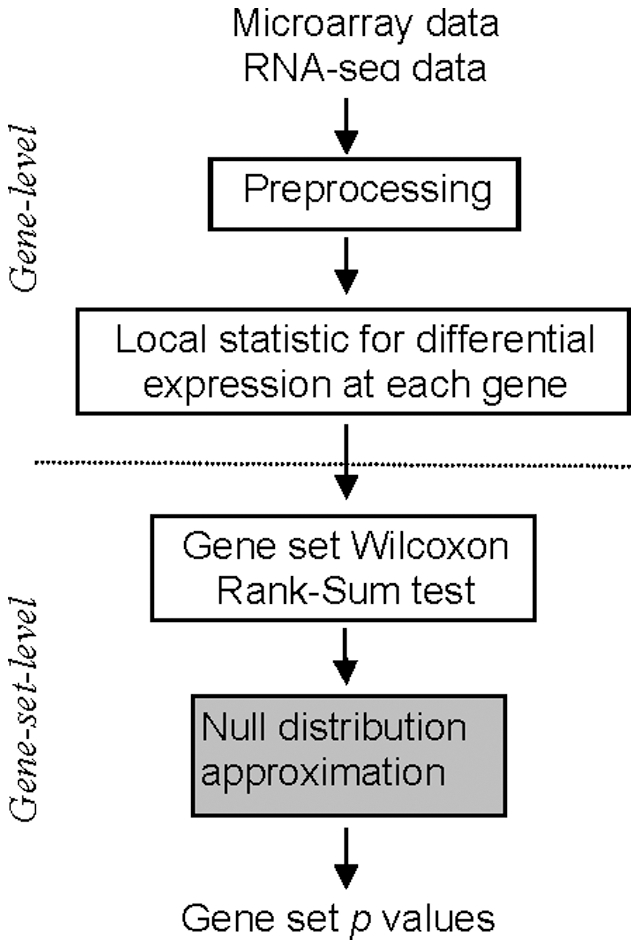
Flowchart for the analysis of real data. The location for null distribution approximation is highlighted with grey background.

As shown in **Figure 5**, we first conducted data preprocessing for each real data set. Gene-level test statistic is then calculated for each gene. The gene-level test statistics were used to compute the WRS statistic for each GO term by comparing the genes in the GO term with the genes in the data set but outside of the GO term. A p value was obtained for each GO term based on the null distribution from a normal or uniform approximation.

*Marioni Microarray dataset* was generated for the comparing gene expressions in human liver and kidney tissues [19]. Three Affymetrix Human Genome U133 Plus 2.0 arrays were used to profile gene expressions in liver and kidney samples separately. The microarray data were downloaded from Gene Expression Omnibus (GEO) with accession number GSE11045.

*Song microarray dataset* was downloaded from GSE7869. Twenty one Affymetrix Human Genome U133 Plus 2.0 arrays were used to profile gene expressions on cysts of different sizes and minimally cystic tissue (MCT) from five PKD1 (polycystic kidney disease) human polycystic kidneys, and non-cancerous renal cortical tissue from three nephrectomized kidneys with isolated renal cell carcinoma [20]. They found that all cyst samples consistently clustered as a single group (13 samples), while the MCT and normal renal cortical samples clustered as a second group (8 samples). We compare these two groups for our gene set analysis.

After downloading the microarray datasets, we preprocessed the raw data using the robust multiple-array analysis (RMA) procedure [21] with quantile normalization (function “*rma*” in the Bioconductor package “*affy*”). The Affymetrix feature names were mapped to Ensembl gene IDs using Bioconductor package *biomaRt*. The expression level for each Ensembl gene ID was calculated as the median of expression values (on log2 scale) corresponding to the Affymetrix probe sets mapped to the same Ensembl gene ID. Two-sample *t* statistic or simple mean fold change was calculated as the local statistic for each gene in microarray datasets.

*Marioni RNA-seq dataset* was generated using the Illumina genome analyzer with each RNA sample being sequenced in seven lanes, of which five lanes were at 3 pM concentration and two lanes at 1.5 pM concentration. The RNA-seq expression file was obtained from the supplemental Table 2 in [19]. We only used the 3 pM concentration lanes from liver and kidney. We first filtered out genes not expressed in both liver and kidney samples. This results in 22490 genes remaining for analysis.

*Kim RNA-Seq dataset* was also generated using the Illumina genome analyzer [22]. RNA samples from 3 biological replicates of pancreatic islets were compared between normal female and pregnant female mice. The aligned data, after mapped to the Refseq collection of mouse transcripts from mouse genome build MM9/2007, were downloaded through GSE21860. For quantifying gene expression, we discarded reads mapping to multiple transcripts or having more than two mismatches.

For each gene in the RNA-seq datasets, a Wald-type statistic was calculated as the local statistic. For example, if x, y are the sums of sequence counts for a given gene in the liver and kidney samples in the Marioni RNA-seq dataset, then the local statistic for this gene is 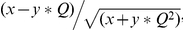 where 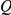 is the ratio of the sum of total numbers of mapped reads in the two tissues. For comparison, we also adopt likelihood ratio test statistic, under the assumption of Poisson model or negative binomial model, as the local statistic. LR statistics were obtained using R functions *glmFit()* and *glmLRT()* in Bioconductor package *edgeR* [23]. We used the common dispersion computed by the function *estimateCommonDisp()*. GO terms mapped to Ensembl genes or RefSeq genes were extracted using Bioconductor package *biomaRt*.
